# Advanced Strategies and Mechanisms of Nanomaterial–Molecularly Imprinted Polymer Synergistically Functionalized Biosensors for Biomarker Detection

**DOI:** 10.3390/bios16050257

**Published:** 2026-05-01

**Authors:** Yaru Zhang, Tao Zhao, Chaoyun Li, Yong Huang

**Affiliations:** 1 College of Pharmacy, Guangxi Medical University, Nanning 530021, China; 202321064@sr.gxmu.edu.cn (Y.Z.); 202421041@sr.gxmu.edu.cn (C.L.); 2 State Key Laboratory of Targeting Oncology, Guangxi Medical University, National Center for International Research of Bio-Targeting Theranostics, Guangxi Medical University, Guangxi Key Laboratory of Bio-Targeting Theranostics, Guangxi Medical University, Collaborative Innovation Center for Targeting Tumor Diagnosis and Therapy, Guangxi Medical University, Guangxi Talent Highland of Major New Drugs Innovation and Development, Guangxi Medical University, Targeting Theranostics Research Center of Guangxi Higher Education, Guangxi Medical University, Nanning 530021, China; zhaotao@sr.gxmu.edu.cn

**Keywords:** molecularly imprinted polymer (MIP), electrochemical biosensor, nanomaterials, biomarker detection, signal amplification, point-of-care testing (POCT)

## Abstract

Biomarker detection demands low cost, rapid turnaround, interference resistance, and wide dynamic range. However, traditional immunoassays and nucleic acid amplification methods remain constrained by complex matrices, batch stability, and portability limitations. Molecularly imprinted polymers (MIPs) exhibit “artificial antibody”-like specific recognition and high stability, while nanomaterials (NMs), depending on their composition, structure, and interfacial organization, can provide conductive pathways, catalytic activity, high-density loading sites, or mass-transfer-favorable architectures. Electrochemical biosensors synergistically constructed from these two components achieve complementary functions in recognition, mass transfer, and signal transduction. This paper systematically reviews key strategies and mechanisms for NM–MIP synergistic construction, focusing on six synergistic strategies that target key bottlenecks in mass transfer, signal generation, and interfacial stability: dynamic response regulation, hierarchical structural engineering, anti-fouling interfaces, multi-signal cross-validation, catalytic–recognition integration, and interfacial binding regulation. Representative biomarker cases are analyzed to illustrate how functional modules can coordinate across sample processing, signal generation, and recognition confirmation to improve analytical reliability and overall sensing performance. Finally, the review discusses challenges in clinical translation, including consistent manufacturing, matrix interference, long-term stability, and standardized validation, while outlining future directions toward mechanism-guided imprint design, intelligent data-assisted optimization, and integration with microfluidic and wearable platforms for multiplexed biomarker detection.

## 1. Introduction

Biomarkers refer to molecular, cellular, tissue, or imaging indicators that quantitatively or qualitatively reflect normal physiological processes, pathological changes, or drug responses in the body. They hold significant value in early disease diagnosis, disease progression monitoring, prognosis assessment, and treatment efficacy evaluation, with widespread application particularly in the early diagnosis and personalized treatment of cancer, cardiovascular diseases, and immune-related disorders [1,2,3].

With the rapid advancement of precision medicine, clinical demands for biomarker detection have intensified (lower cost, shorter turnaround time, stronger interference resistance, and broader dynamic range). However, mainstream methods such as enzyme-linked immunosorbent assay (ELISA), chemiluminescent immunoassay (CLIA), and polymerase chain reaction (PCR) are often limited by factors like antibody/probe batch variability, susceptibility to inactivation, and high preparation costs. These methods frequently exhibit restricted sensitivity and dynamic range, carry a higher risk of false positives, and demand strict experimental environments and operational protocols. Their complex sample pretreatment workflows make them ill-suited to meet the “rapid, simple, low-cost” requirements for major disease screening and point-of-care testing (POCT) [4,5,6,7]. Although mass spectrometry techniques (e.g., LC-MS/MS) offer high specificity and simultaneous multi-component detection for metabolite and protein analysis, their high equipment and testing costs, lengthy turnaround times, and complex pretreatment procedures limit their application in primary care and large-scale screening [8,9]. Consequently, developing novel detection platforms that combine high sensitivity, high selectivity, low cost, and portable potential has become a critical research direction in precision medicine.

Electrochemical biosensors convert biological recognition events into quantifiable electrical signals, offering advantages such as rapid response, lower cost, and ease of miniaturization and portability. They can cover the detection of various biomarkers, including nucleic acids, proteins, and metabolites [10,11,12], and possess potential for POCT development. However, non-specific adsorption and coexisting interferences in complex biological matrices, insufficient long-term stability of sensing interfaces, and stringent requirements for sensitivity and batch-to-batch consistency in detecting ultra-low-abundance markers still significantly constrain their clinical translation.

Against this backdrop, electrochemical biosensing strategies synergistically integrating molecularly imprinted polymers (MIPs) and nanomaterials (NMs) have garnered extensive attention. MIPs, often termed “artificial antibodies,” can form three-dimensional recognition cavities within polymers through molecular imprinting technology (MIT). These cavities complement the shape, size, and functional group distribution of target molecules, enabling antibody-like specific capture [12]. Compared to natural antibodies, MIPs typically offer higher environmental stability, lower cost, and greater scalability for batch synthesis. Depending on their material class, morphology, redox state, and interfacial assembly, nanomaterials can enhance sensing performance by providing conductive pathways, catalytic activity, hierarchical transport architectures, or enrichment/separation capability. The functional complementarity between these two components—achieved through “specific recognition–signal amplification–interface optimization”—significantly improves detection performance and expands application scenarios.

Accordingly, this review presents a systematic, mechanism-oriented analysis of the synergistic construction of nanomaterial–molecularly imprinted polymer (NM–MIP) systems for biomarker-related electrochemical sensing, with particular emphasis on six representative synergistic strategies: hierarchical structural construction, anti-fouling interfacial regulation, multi-signal cross-validation, catalytic–recognition integration, regulation of interfacial bonding strength, and dynamic-response design. Unlike existing reviews that primarily categorize nanomaterials, optimize electrochemical synthesis and electropolymerization routes, or summarize specific applications, this work emphasizes the synergistic coupling mechanisms among functional material units. In particular, it analyzes the structure–property correlations among nanostructures, imprinted recognition cavities, and electrochemical signal transduction pathways. Dong et al. [13] summarized advances in nanomaterial-modified molecularly imprinted electrochemical sensors, highlighting the roles of nanomaterials in improving conductivity and effective surface area. Ayankojo et al. [14] focused on electrosynthesized imprinted sensors for medical diagnostics, emphasizing the advantages of electrochemical fabrication in controlling film thickness, morphology, and interfacial properties. Ramajayam et al. [12] reviewed the expansion of molecular imprinting strategies to diverse sensing platforms from a biomimetic materials perspective. Although these reviews discuss the coupling of nanomaterials with imprinted recognition units, they generally lack a unified organizational framework centered on synergistic mechanisms and functional modules that integrate the key bottlenecks of mass transfer, charge transport, and interfacial stability and explicitly link them to their underlying mechanisms. The review is centered on electrochemical biosensing platforms, and the studies discussed were selected primarily for their relevance to synergistic mechanisms and functional coupling. At the same time, to further illustrate issues related to imprinted-interface design, signal transduction behavior, and cross-validation logic, a limited number of studies employing optical readouts, such as resonant light scattering (RLS) and fluorescence, are also included where appropriate.

**Figure 1:**
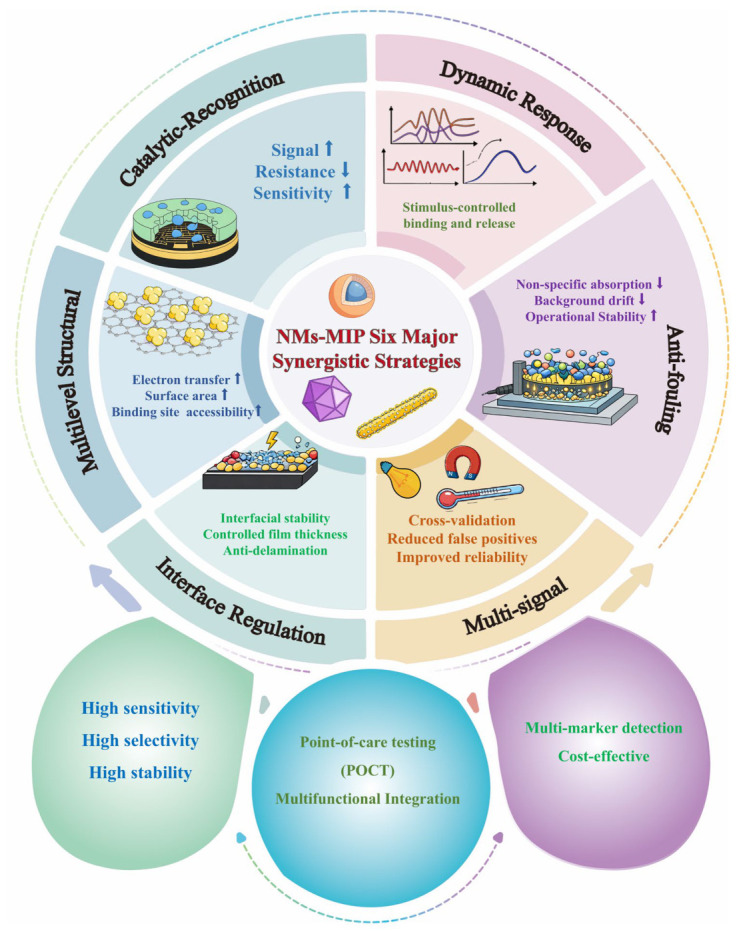
Six major synergistic strategies for nanomaterial–molecularly imprinted polymer (NM–MIP) electrochemical biosensors.

On this basis, we first examine how nanomaterials and imprinted polymer layers are assembled at electrochemical interfaces and analyze how such assembly affects interfacial stability, imprint-layer thickness, and the accessibility of recognition sites. We then discuss how recognition events are translated into measurable electrochemical outputs through changes in interfacial impedance, regulation of charge transfer, and modulation of diffusion behavior. From this perspective, the six strategies are further considered in terms of their distinct roles in governing mass transfer, signal generation and readout reliability, and interfacial stability. It should be noted that these six strategies do not follow a single uniform classification logic: some mainly reflect direct functional synergy between nanomaterials and imprinted layers, whereas others more strongly represent the structural or interfacial features that facilitate synergy or the responsive modes through which synergistic behavior is regulated. By integrating representative biomarker-detection studies, this review further compares the applicability, stability, and response characteristics of different strategies in complex matrices, with the aim of establishing clearer links among material design, interfacial regulation, and signal output. In doing so, it seeks to provide a transferable analytical framework for the structural optimization and performance enhancement of NM–MIP systems, while also offering a useful reference for shifting related research from material stacking toward mechanism-driven design. A schematic overview of the six representative synergistic strategies discussed in this review is presented in Figure 1.

## 2. Core Technological Foundations: Integration of “Individual Strengths”

### 2.1. MIT: Customizable “Artificial Antibodies”

#### 2.1.1. Development and Principles of Molecular Imprinting

MIT originated in the 1930s, when Polyakov observed specific adsorption phenomena in silica gel studies [15]. Subsequently, Pauling’s antigen–antibody complementarity theory provided crucial insights for MIT [16,17]. In the 1970s, Wulff’s team pioneered covalent imprinting [18], while Mosbach proposed non-covalent imprinting systems in the 1980s [19]. In 1995, the Whitcombe group introduced a semi-covalent imprinting strategy [20], collectively establishing the theoretical and methodological foundations of MIT. Entering the 21st century, surface imprinting [21,22], controlled active polymerization [23,24], green synthesis [25], 3D printing [26,27], and AI-assisted design [28] propelled MIT toward high performance, intelligence, and manufacturability.

MIT employs the “lock-and-key” recognition principle, using template molecules to induce the formation of molecularly imprinted polymers (MIPs) with specific recognition capabilities. The typical preparation process comprises three steps: template–monomer preassembly, crosslinking polymerization and curing, and template elution [29]. During pre-assembly, the template forms covalent or non-covalent interactions with functional monomers to create complexes [30,31,32]. Subsequently, under the action of crosslinkers and initiators, a rigid three-dimensional network is formed [33,34,35]. Finally, solvent extraction or chemical treatment removes the template, yielding recognition cavities complementary to the template and enabling highly selective binding [36,37,38].

#### 2.1.2. Molecular Imprinting Preparation Methods

MIP preparation methods can be categorized into three types based on material morphology and polymerization strategy:

(1) Conventional bulk polymerization: Bulk polymerization offers simple operation but requires grinding, which can damage cavity structures, and template elution efficiency is typically low (usually <60%). Phase inversion polymerization directly yields films/microspheres without grinding, but morphology and pore size distribution are highly solvent-dependent [29,39].
(2) Nanoscale Polymerization Methods: To enhance mass transfer efficiency and accessibility of recognition sites, nanoscale polymerization methods have been extensively developed. Precipitation polymerization yields relatively uniform submicron particles without surfactants, but binding site density is influenced by template concentration [40,41]; emulsion polymerization enables size-controlled nanoscale MIPs but carries risks of surfactant residue [38]; Core–shell polymerization integrates recognition and signal output by constructing MIP shells on functional nanoparticle surfaces [37,42]; solid-phase polymerization enhances reproducibility and reduces template leakage through template immobilization, making it suitable for large-scale production [30,43].
(3) Surface imprinting: This method constrains recognition sites to material surfaces, suitable for macromolecular templates [44,45]. Sacrificial carrier methods improve elution and kinetics, while soft photolithography constructs micro/nano recognition structures for microbial capture [46]. Electropolymerization enables in situ formation of MIP films on electrode surfaces, achieving “one-step” sensor fabrication [47]. Notably, studies have employed epitope imprinting to utilize protein-characteristic peptides as templates, enhancing recognition of intact proteins while reducing conformation sensitivity [48,49].

### 2.2. Application of Nanomaterials in Molecularly Imprinted Electrochemical Sensors

Nanomaterials are widely used in molecularly imprinted electrochemical sensors because different material classes can contribute distinct functions, including conductive support, catalytic amplification, porous transport channels, high-density loading sites, and magnetic enrichment/separation. These effects depend strongly on material type, oxidation/redox state, morphology, and assembly mode. For example, metal nanoparticles (AuNPs, PtNPs) can promote charge transfer and catalytic reactions [50,51,52,53,54]; carbon-based materials, including graphene-derived materials and carbon nanotubes, can serve as conductive scaffolds and immobilization platforms, although their effective conductivity and accessible surface area depend on oxidation state, defect density, and interfacial assembly [55,56,57,58]; metal–organic frameworks (MOFs) and covalent organic frameworks (COFs) can provide porous structures for target capture, enrichment, and catalytic amplification [59,60,61,62]; MXene-based materials can offer conductive layered supports with good surface functionalizability [63,64]; and magnetic nanomaterials (Fe_3_O_4_) can enable magnetic separation and pre-enrichment in complex samples [65,66]. Multimaterial composites can further provide functional complementarity, thereby improving overall detection performance [67]. NM–MIP electrochemical biosensors demonstrate high sensitivity and selectivity in detecting biomarkers, drugs, and pollutants, exhibiting significant application potential in biomedicine and environmental monitoring [68,69,70].

### 2.3. Electrochemical Biosensing: The “Information Decoder” for Biological Recognition

Electrochemical biosensors enable quantitative detection by converting recognition events into electrical signals. Recognition elements may include enzymes, antibodies, nucleic acids, aptamers, or biomimetic MIPs [71,72]. The interface redox processes, charge distribution, or impedance changes triggered by recognition are transduced to output current (current-based) [73,74], potential (potential-based) [75,76], or impedance (impedance-based) [77,78,79,80]. Electrochemical biosensing offers high sensitivity, rapid response, and miniaturization advantages, demonstrating significant potential in point-of-care diagnostics and environmental monitoring [81,82]. Current research focuses on enhancing sensor stability [83,84], improving interference resistance in complex samples, and developing multifunctional integrated platforms [85,86,87,88].

## 3. Strategies and Mechanisms for Synergistic Modification of Electrochemical Biosensors Using NM–MIP

Molecularly imprinted polymers (MIPs) offer advantages such as high stability, structural tunability, and low cost; however, when used at electrochemical interfaces, they still commonly suffer from non-uniform film morphology, limited interfacial electron-transfer efficiency, deeply buried recognition sites, and nonspecific adsorption. These limitations not only reduce recognition efficiency and signal responsiveness but may also compromise analytical stability and practical applicability in complex matrices. By contrast, nanomaterials can provide conductive pathways, hierarchical mass-transfer architectures, enrichment or separation capability, and signal-amplification effects, thereby helping to address the limitations of MIPs in interfacial construction, mass-transfer regulation, and signal transduction, and thus contributing to improvements in the sensitivity, selectivity, and application potential of the overall system.

**Figure 2:**
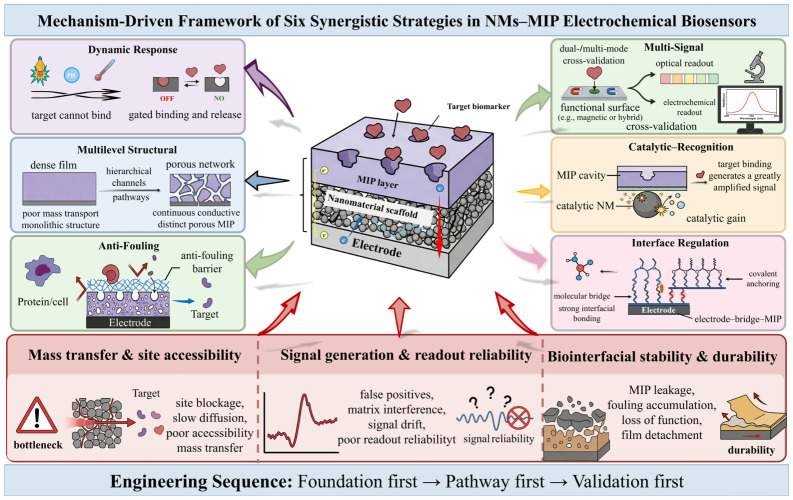
Mechanism-Driven Framework of Six Synergistic Strategies in NM–MIP Electrochemical Biosensors.

Against this background, the following sections discuss six representative synergistic strategies. Although these strategies differ in their primary modes of action, they can contribute, to varying extents, to the optimization of several key issues relating to electrochemical sensing interfaces, particularly mass transfer and site accessibility, signal generation and readout reliability, and interfacial stability. By examining representative studies, this section further highlights the principal role of each strategy in performance enhancement, as well as the contexts in which it may be most effectively applied. The mechanism-oriented organization of these six synergistic strategies is illustrated in Figure 2.

### 3.1. Dynamic Response Synergy: Stimulus-Gated Imprinted Interfaces for Controllable Binding and Release

Dynamic response synergy is achieved by introducing stimulus-responsive units, such as light, magnetic fields, pH, or temperature, so that the imprinted sites undergo reversible conformational and affinity changes under external stimuli, enabling controllable binding and release. At the same time, nanomaterials can contribute functions such as enrichment and separation, surface-area expansion, improved site accessibility, and signal carriage, complementing the stimulus-responsive imprinted network. The former improves operability and compatibility with complex matrices, whereas the latter enhances regenerability and reusability, and together they improve the practical performance of the sensing system. It should also be noted that, although some examples of stimulus-gated NM–MIP systems employ optical signal readouts such as resonant light scattering (RLS) or fluorescence, the underlying material and interfacial design concept—namely, gating of imprinted sites and regulation of interfacial permeability—is equally relevant to electrochemical sensing interfaces. Changes in site openness and interfacial permeability often affect charge-transfer processes and, consequently, signal output. This strategy mainly improves controllability and reusability by enabling stimulus-gated regulation of binding, release, and interfacial permeability.

#### 3.1.1. Fe_3_O_4_@SiO_2_@MOF Magnetic Core–Shell + Photoresponsive Monomer: Synergy of Light-Controlled Recognition and Magnetically Controlled Enrichment

Wang et al. [89] developed a dually photo- and magnetically responsive molecularly imprinted system targeting enterovirus 71 (EV71) using Fe_3_O_4_@SiO_2_@UiO-66-NH_2_-C=C as the magnetic carrier and the azo-based photoresponsive functional monomer AOPBA (4-(4′-acryloyloxyphenylazo)benzoic acid). In this system, the Fe_3_O_4_ magnetic core primarily enables rapid magnetic separation and enrichment during sample processing. Meanwhile, the core–shell structure and the metal–organic framework (MOF) component UiO-66-NH_2_ provide a large specific surface area and a stable loading platform, which helps increase imprinted-layer loading and improve site exposure. AOPBA undergoes reversible trans–cis isomerization under UV and visible light, thereby modulating the binding strength between the imprinted layer and the virus and enabling a photochemical switch between adsorption and release. The study showed that reversible adsorption/release transitions could be achieved under 365 nm UV light and visible-light irradiation. In addition, a no-magnetic-separation control experiment produced nearly identical light-controlled responses, indicating that magnetic separation mainly improved operational efficiency without compromising the photoresponsive recognition behavior of the system. The method showed a linear range of 0.05–5.0 U/mL for EV71, a detection limit of 9.5 × 10^−3^ U/mL (3.9 fM), and an imprinting factor of 5.1. Recoveries in 100-fold diluted human serum were 94.0–108.4%. The authors further reported that the sensing material retained about 90% of its photoresponse signal after 6 months of storage and that the incorporation of the magnetic core shortened the adsorption equilibrium time to within 20 min. This example shows that magnetic nanostructures can provide rapid separation and process controllability, while photoresponsive monomers impart reversible recognition and regeneration. Their combination enhances both compatibility with complex samples and reusability.

#### 3.1.2. MIL-101-Supported pH-Responsive MIP: Synergy of Acid-Triggered Swelling for Elution/Regeneration and Mass-Transfer Regulation

Luo et al. [90] reported a pH-responsive molecularly imprinted nanoprobe, HM@MIPs, based on the MOF material MIL-101 for hepatitis A virus (HAV) detection, with RLS as the signal readout. In this system, the pH-responsive imprinted layer was constructed on the surface of MIL-101. Its high specific surface area provides more available sites for imprinting and a higher adsorption capacity, and comparison with a SiO_2_ substrate was used to evaluate the contribution of a high-surface-area support to viral imprinting and sensing performance. The pH-responsive behavior of the imprinted layer was examined systematically under different pH conditions. The results showed pronounced swelling under acidic conditions, which was attributed to protonation of dimethylaminoethyl methacrylate (DMA) units in acidic media, leading to increased polymer hydrophilicity and network swelling. Importantly, the optimal pH for detection in this work was 8.0, with an incubation time of 20 min, whereas the optimal pH for template elution was 2.0. Thus, the pH response in this system is more appropriately understood as acid-triggered swelling that promotes template elution/regeneration and improves mass-transfer processes, rather than as stronger binding under acidic conditions. Under optimal conditions, the RLS signal was linear over 0.02–2.0 nmol/L, with a detection limit of 0.1 pmol/L. Recoveries in 200-fold diluted human serum were 88.0–108.0%, with RSD values of 1.8–2.5%. This case shows that MOFs can provide high site density and site accessibility, while pH-responsive networks offer a mild and controllable regeneration route. Their combination improves the usability and reusability of virus-imprinted probes in complex matrices.

#### 3.1.3. CD@SiO_2_-PNIPAAm Thermoresponsive Platform: LCST-Regulated Binding–Release Synergy

Zhao et al. [91] used silylated carbon-dot composites (CD@SiO_2_) as fluorescent signal carriers and introduced N-isopropylacrylamide (NIPAAm) to construct a thermoresponsive molecularly imprinted fluorescent sensing material (CD@SiO_2_@MIP) for bovine hemoglobin (BHb) detection. In this system, polymerization of NIPAAm generated poly(N-isopropylacrylamide) (PNIPAAm) segments with a lower critical solution temperature (LCST) of about 32 °C. CD@SiO_2_ served as both a stable signal source and a dispersive support, whereas the PNIPAAm segments conferred temperature responsiveness and acted as a temperature switch for controlling template capture and release. Above the LCST, the PNIPAAm segments become more hydrophobic, and the adsorption/binding response decreases, whereas below the LCST they become more hydrophilic and the response increases, enabling reversible thermo-regulated capture and release. The sensor showed a linear range of 0.31–15.5 μM, a detection limit of 0.155 μM, and an imprinting factor of 3.11. In 100-fold diluted urine samples, recoveries for BHb were 98.6–100.5%, with RSD values of 0.85–2.6%. This case demonstrates that signal-active nanomaterials can provide measurable signal output and material stability, whereas stimulus-responsive imprinted networks offer controllable regeneration and reuse. Their combination improves operational convenience and adaptability to real samples without sacrificing recognition selectivity.

### 3.2. Multilevel Structural Synergy: Coupled Construction of Hierarchical Mass-Transfer Channels and Conductive Networks

Conventional MIP films formed on electrode interfaces often suffer from dense film structures, limited pore diversity, and insufficient effective surface area. These features prolong analyte diffusion pathways, reduce site accessibility, and increase interfacial charge-transfer resistance. Multilevel structural synergy addresses these limitations by incorporating nanomaterials with distinct dimensional or structural features, such as one-dimensional tubular architectures, 0D–2D hybrid structures, and layered metal oxide nanocrystals. In this way, site exposure and mass-transfer efficiency are improved at the structural level, while more continuous conductive pathways for electron transport are established at the electrical level, ultimately enhancing both response speed and sensitivity. This type of structural design primarily improves sensing performance by increasing site accessibility, shortening diffusion pathways, and supporting more continuous conductive networks.

#### 3.2.1. Soft-Template-Induced PPy–MO Rectangular Nanotubes: Synergy of Morphology Regulation and Charge Transfer

Taheri et al. [92] used fluorine-doped tin oxide (FTO) electrodes as substrates to construct a dual-template molecularly imprinted electrode for label-free impedimetric detection of the lung cancer markers alpha-fetoprotein (AFP) and carcinoembryonic antigen (CEA). A key feature of this design was the introduction of methyl orange (MO) during the electropolymerization of pyrrole (Py). Py monomers interact with the sulfonic acid groups of MO, and polymer growth is initiated around the MO template, giving rise to polypyrrole–methyl orange (PPy–MO) nanotube morphologies. The authors noted that nanotubular structures generally exhibit better binding affinity, more favorable electrochemical properties, and higher adsorption capacity than other morphologies, which in turn improves rebinding efficiency and signal response. Electrochemical characterization further showed that the FTO/PPy–MO layer had higher conductivity than bare FTO, indicating that the PPy conductive network lowered electron-transport resistance. At the same time, MO acted as a functional unit that regulated both morphology and charge transfer, producing a stable and highly conductive imprinted interface. The sensor achieved detection limits of 3.3 pg/mL for AFP and 1.6 pg/mL for CEA, with linear ranges of 10–10^4^ pg/mL for AFP and 5–10^4^ pg/mL for CEA. Recoveries in spiked samples were 92.0–94.2% for AFP and 96.0–98.8% for CEA. This case suggests that soft templates or dopants such as MO do more than generate porous structures; they can simultaneously regulate morphology and conductive/charge-transport behavior, bringing imprinting sites closer to accessible interfaces and reducing background loss in impedance-based readout.

#### 3.2.2. 0D–2D Carbon Dot/Graphene Point–Surface Composite: Synergy of Dispersion and Conductive Support

Pakapongpan et al. [93] developed a nitrogen-doped carbon dot (NCD)–graphene nanocomposite support for Aβ42 detection and electropolymerized a PPy–MIP recognition layer on its surface. The introduction of NCDs helped suppress aggregation of graphene sheets and improved their dispersion, thereby facilitating the formation of a stable and uniform interface. At the same time, the NCD–graphene composite increased the effective surface area, conductivity, and electrocatalytic activity of the platform, as reflected by an approximately twofold increase in peak current in cyclic voltammetry (CV). These combined effects promoted the construction of a more efficient interface for electron transfer and signal output. The sensor exhibited a linear range of 5–70 pg/mL, a detection limit of 1 pg/mL, and reproducibility with an RSD of 2.08%. This example shows that the value of 0D–2D composites extends beyond conductivity enhancement alone. Equally important is the role of 0D nanoparticles as spacers and dispersants that help stabilize the 2D network, prevent interfacial collapse or inhomogeneity, and support the formation of a reproducible recognition–conduction coupling interface for thin MIP films.

#### 3.2.3. Layered MOx Nanocrystals Embedded in the Imprinted Layer: Synergy of Enhanced Electron Transfer and Tunable Sensitivity

Saddique et al. [94] incorporated layered metal oxide (MOx) nanocrystals, including MoO_3_, V_2_O_5_, and MoO_3_:V_2_O_5_ (schlegelite), at different loadings into poly(MAA-co-EGDMA)-based imprinted polymers to form composite receptor layers on disposable screen-printed gold electrodes for creatinine detection. Cyclic voltammetry showed that, compared with pure MIP, the sensitivities of MIP/MoO_3_, MIP/V_2_O_5_, and MIP/MoO_3_:V_2_O_5_ increased by 113%, 121%, and 319%, respectively. The Schlegelite system exhibited a detection limit of 90 nM, a response time of <1 min, and a linear range of 5–30 μM, which the authors noted corresponds well to practical saliva concentrations. The study further indicated that the introduction of an inorganic phase increased sensitivity and lowered the detection limit to the nanomolar level, while the transition-metal oxides improved electron transfer during electrochemical processes and contributed to greater stability and durability. The authors also discussed the possible hard acid–base affinity between the metal oxides and the –NH_2_ groups of creatinine, which may further strengthen binding and enhance signal intensity. This example suggests that the key role of layered MOx nanocrystals lies in the dual contribution of enhanced electron transfer and increased binding/adsorption affinity. By tuning the type and loading of the inorganic phase, sensitivity and working range can be engineered without relying solely on thicker or denser imprinted layers.

### 3.3. Anti-Fouling Synergy: Constructing Hydrophilic/Barrier Interfaces to Suppress Non-Specific Adsorption

In complex matrices such as serum, urine, and even whole blood, proteins, cells, and small molecules coexist and readily cause non-specific adsorption at electrode interfaces, resulting in baseline drift, signal attenuation, and reduced reproducibility. Anti-fouling synergy typically suppresses contamination by introducing hydrophilic or charged terminal groups or physical barrier layers, either outside the recognition layer or at critical interfacial regions. At the same time, highly conductive or high-surface-area nanomaterials help preserve interfacial charge transfer and mass transport, thereby maintaining sensing performance while improving fouling resistance. Successful operation of a sensor in real complex samples can provide preliminary evidence of its anti-fouling capability; where feasible, this should be further validated using quantitative metrics such as signal retention, signal-suppression rate, the interference-to-target signal ratio, and comparisons between responses obtained in buffer and in real samples.

#### 3.3.1. LSG/AuNPs Porous Conductive Underlayer + PEDOT–MIP: Synergy of Porous Mass Transfer and Anti-Fouling

Ait Lahcen et al. [95] produced laser-scribed graphene (LSG) on polyimide sheets using a CO_2_ laser, electrodeposited gold nanostructures (AuNPs) onto the LSG, and then constructed a poly(3,4-ethylenedioxythiophene) (PEDOT)-based imprinted layer for human epidermal growth factor receptor 2 (HER-2) detection. The three-dimensional porous conductive framework of LSG increased the effective reaction area and promoted both mass transfer and charge transfer. AuNPs further increased conductivity and surface area and facilitated HER-2 adsorption prior to MIP formation. The sensor showed good analytical response over 1–200 ng/mL, with a detection limit of 0.43 ng/mL. In spiked undiluted human serum, the sensor showed appreciable recovery values, supporting its applicability in complex matrices. Accordingly, this case indicates that porous conductive scaffolds help maintain signal transmission in undiluted serum. The study also reported the background response of unspiked serum and explicitly noted that this signal could arise either from endogenous Her-2 in serum or from the nonspecific adsorption of serum proteins on the sensor surface. This suggests that, although this architecture offers practical advantages for operation in undiluted serum, the respective contributions of background interference and true target recognition still require clearer quantitative differentiation. However, the original study did not further dissect these contributions quantitatively. In this sense, the present case can be regarded as a representative example of applicability in complex matrices. However, if future studies on similar architectures could combine real-sample recovery data with more explicit antifouling validation, such as comparisons between buffer and serum responses, analyses of signal retention after matrix exposure, or protein-adsorption control experiments, the corresponding conclusions would be more convincing.

#### 3.3.2. rGO/PDA Hydrophilic Adhesive Substrate: Synergy of Multi-Marker Anti-Fouling and High Loading

Li et al. [96] constructed a reduced graphene oxide (rGO)/polydopamine (PDA) composite substrate and prepared a multi-marker MIP using creatinine, urea, and human serum albumin (HSA) as template molecules, enabling simultaneous detection of multiple renal disease biomarkers in serum and urine. The key feature of this design was a surface molecular imprinting strategy that formed a thin PDA-MIP layer in situ on the rGO surface. As a conductive two-dimensional scaffold, rGO not only improved conductivity and electron-transfer efficiency but also increased the effective surface area and MIP loading capacity, while helping to avoid the template entrapment problems associated with thick conventional MIP layers. In addition, this surface-MIP design avoids the deep embedding associated with excessively thick traditional MIP layers and was further validated in serum and urine samples. The platform achieved femtomolar detection limits for all three biomarkers, and interference studies showed only minor signal perturbation from common coexisting substances. More specifically, for creatinine and urea, the maximum interferent response was reported to be 9.6% of the target signal, while for HSA, the responses to myoglobin and cytochrome c were below 8.0%, although higher cross-responses were observed for lysozyme and avidin. In addition, the clinical sample results remained within 81.8–119.1% of the hospital measurements, with an RSD below 7.9%. More importantly, the method was validated against clinical test results in real human urine and serum samples, demonstrating the practicality and anti-interference capability of this interfacial design under complex matrix conditions. This case suggests that anti-fouling and matrix-tolerant design do not necessarily require an additional dedicated anti-fouling overlayer. A practical alternative is the combination of thin-layer surface imprinting with a conductive support, which may help reduce non-specific retention associated with thick films while preserving signal output. Additionally, to provide more convincing evidence of interference resistance, one can include representative interference spectra and quantitative metrics such as the interference-to-target signal ratio for verification.

#### 3.3.3. SA-Doped Hydrophilic Surface-Imprinted Interface: Hydration-Layer-Assisted Suppression of Non-Specific Adsorption

Yang et al. [97] developed an anti-fouling surface molecularly imprinted ratiometric electrochemical biosensor for SARS-CoV-2 spike protein by incorporating sodium alginate (SA) into a PPy-based imprinted layer on a boronate-affinity AuNPs/SPCE interface. In this design, SA served not merely as a co-dopant but as a hydrophilic interfacial component that helped suppress nonspecific adsorption while preserving signal readout through a ratiometric iron phthalocyanine (PcFe)/[Fe(CN)_6_]^3−/4−^ scheme. The analytical platform showed a wide linear range from 100 fg mL^−1^ to 100 ng mL^−1^, with a low detection limit of 30.1 fg mL^−1^. More importantly, the anti-fouling role of SA was supported by direct comparative evidence. In interference tests, biosensors fabricated without SA exhibited more noticeable signal changes in the presence of coexisting species, whereas SA-containing interfaces showed reduced nonspecific response. This trend was further corroborated by fluorescence microscopy: obvious adsorption of HeLa cells was observed on the surface without SA, while little to no obvious cell adsorption was seen after SA incorporation. Contact-angle measurements also supported the proposed mechanism, with the water contact angle decreasing from 52° for bare SPCE to 31° for the SMI layer without SA and further to 10° after SA doping, consistent with enhanced surface hydrophilicity and hydration-layer formation. In addition, the sensor was successfully applied to clinical serum samples by standard addition, giving recoveries of 98.9–103.3% with RSD values below 5%. Taken together, this case provides relatively direct support for the view that introducing highly hydrophilic components into a surface-imprinted interface can mitigate nonspecific adsorption in protein-containing environments while maintaining reliable electrochemical detection. At the same time, because the real-sample validation was based on serum spiking rather than a broader fouling metric framework, the anti-fouling claim is better interpreted as being substantially strengthened, rather than exhaustively established.

#### 3.3.4. MOF Functional Layer + PTB Top Barrier: Mass Transfer/Anti-Fouling Synergy for Direct Whole-Blood Detection

Zhu et al. [98] constructed a PTB/MIP/Ni_3_(HITP)_2_-MOF/SPCE system for direct glucose detection in whole blood. In this architecture, Ni_3_(HITP)_2_-MOF served as a conductive MOF intermediate layer that significantly enhanced interfacial electron transfer. Phase-transition bovine serum albumin (PTB), formed by tris(2-carboxyethyl)phosphine (TCEP)-induced reduction in BSA disulfide bonds followed by amyloid-like aggregation, acted as a protein nanomembrane top layer with both strong anchoring capability and anti-fouling function, suppressing signal attenuation caused by non-specific adsorption in whole blood. Whole-blood adsorption experiments showed that the signal of the electrode without PTB decreased markedly as whole-blood concentration increased, whereas the PTB-modified electrode showed less than 5% signal suppression even after treatment with 100% whole blood. Its anti-protein-fouling effect was further supported by adsorption control experiments using fluorescein isothiocyanate-labeled bovine serum albumin (FITC-BSA). Strong fluorescence accumulation was observed on SPCE and MIP/Ni_3_(HITP)_2_-MOF/SPCE, whereas the PTB/MIP/Ni_3_(HITP)_2_-MOF/SPCE interface showed no obvious fluorescence increase over time, indicating effective suppression of nonspecific protein adsorption. The sensor exhibited a linear range of 1 μM–100 mM for glucose, with a detection limit of 0.31 μM, allowing direct whole-blood detection without complicated pretreatment. In addition, the sensor was validated in 30 real whole-blood samples, achieving a detection accuracy of 94.2% relative to the hospital gold-standard method, with an RSD below 4.2%, further demonstrating its applicability in highly complex matrices. More broadly, this case suggests a transferable principle for complex-matrix MIP sensing: under heavily fouling conditions, matrix tolerance may be more reliably achieved by functionally separating signal-transmission and contamination-resistance roles within a stratified interface, rather than relying on the imprinted layer alone to fulfill all interfacial tasks.

### 3.4. Multi-Signal Synergy: Dual-/Multi-Mode Cross-Validation for Improved Analytical Reliability

In real samples such as serum, sweat, and urine, single-signal readouts are vulnerable to background autofluorescence, coexisting matrix components, and system fluctuations, which may lead to signal misinterpretation or poor reproducibility. In this context, multi-signal synergy should not be understood simply as the coexistence of multiple outputs but more specifically as the coordinated use of orthogonal functional modules at the levels of sample processing, signal generation, and recognition confirmation to improve analytical reliability. In NM–MIP systems, such designs typically combine separation/enrichment modules, high-contrast or low-background signal-output modules, and secondary recognition modules to integrate selective capture with more reliable readout. On the one hand, magnetic nanomaterials enable rapid enrichment and matrix purification. On the other hand, optical readout modules with high stability, strong enhancement effects, or intrinsically low background, such as persistent luminescence and surface-enhanced Raman scattering (SERS), can improve result reliability by reducing background interference or increasing signal contrast. When a secondary recognition element, such as an aptamer or antibody, is further incorporated, recognition-level cross-validation can be achieved, thereby reducing the risks of false-positive and false-negative results. More broadly, orthogonal hybrid biosensing strategies, including SERS/electrical dual-transduction formats, have likewise shown the value of independent readout pathways for improving analytical confidence [99], although the discussion below remains focused on representative systems more directly related to NM–MIP synergistic design.

#### 3.4.1. Fe_3_O_4_ Magnetic Separation + ZGGC Persistent-Luminescence Readout: Synergy of Enrichment/Purification and Low-Background Quantification

Zhang et al. [100] developed a dual-recognition system combining magnetic molecularly imprinted polymers (m-MIPs) with aptamer-functionalized ZGGC persistent-luminescence nanoparticle probes (ZGGC-Apt) for dopamine (DA) detection without autofluorescence interference. The system used Fe_3_O_4_-COOH as the magnetic carrier, with m-MIPs containing DA template sites prepared on its surface to achieve selective target capture and magnetic separation. Meanwhile, DA aptamers were immobilized on chromium-doped zinc gallogermanate (ZGGC) persistent-luminescence nanoparticles to form ZGGC-Apt as the signal probe. During detection, DA induced the formation of a sandwich complex, m-MIPs@DA@ZGGC-Apt, between m-MIPs and ZGGC-Apt. After magnetic separation removed matrix interference, a quantitative readout was obtained from the persistent luminescence of ZGGC, which greatly reduced interference from sample autofluorescence because no real-time excitation was required during signal acquisition. The method was reported to show a quantitative range of 0.01–100 μM, with a detection limit of 0.0065 μM. In 100-fold diluted real samples (serum, sweat, and urine), DA spiking gave recoveries of 99.2–110.8%, 92.0–112.4%, and 90.6–115.6%, respectively. This case suggests that its synergistic value can be understood in terms of improved analytical reliability at three different levels. At the sample-processing level, the magnetic m-MIPs enable selective capture together with controllable separation, thereby helping reduce matrix interference. At the signal-output level, persistent luminescence provides a low-background readout mode that helps reduce autofluorescence interference because signal acquisition does not require real-time excitation. At the recognition level, the combined use of MIP cavities and aptamers introduces a second level of confirmation beyond single-step interfacial adsorption. Taken together, this system illustrates how reliability may be improved through the coordinated integration of enrichment/purification, low-background signal generation, and secondary recognition. The same platform concept can, in principle, be extended by replacing the template molecule and aptamer.

#### 3.4.2. Fe_3_O_4_-MMIP Capture + Au-SERS Labeling: Synergy of Magnetic Enrichment and Raman Enhancement

Turan et al. [101] developed a magnetic capture–SERS amplification system for prostate-specific antigen (PSA) detection by using magnetic molecularly imprinted nanoparticles (MMIPs) as capture probes and anti-PSA@DTNB@Au as SERS-labeled probes. In this work, an MIP layer was introduced onto Fe_3_O_4_ nanoparticles to enable antibody-free selective binding to PSA, serving as one arm of the sandwich system. The other arm employed DTNB-modified Au nanoparticles to generate a strong SERS signal and was further functionalized with anti-PSA antibodies to enhance specificity in sandwich-complex formation. The detection range was 0.5 pg/mL–1.0 μg/mL, with a linear regression coefficient of R^2^ = 0.996. The detection limit was 0.9 pg/mL, and the quantification limit was 3.2 pg/mL (S/N = 5). In serum samples, recoveries ranged from 98.0% to 101.3%, with RSD values of approximately 3–4%, indicating that the combination of magnetic enrichment and SERS amplification can provide quantitative consistency in complex matrices comparable to reference methods. The synergistic value of this case may be understood at three levels. At the sample-processing level, magnetic MMIPs provide selective capture together with controllable enrichment and separation in serum matrices. At the signal-output level, Au-SERS labels provide a high-contrast optical readout that enables sensitive signal discrimination. At the recognition level, the combination of MMIP-based capture and antibody-functionalized SERS probes adds a second recognition step during sandwich-complex formation. Taken together, this case highlights a transferable design idea for complex-matrix biomarker sensing, in which reliability can be improved by distributing capture/enrichment, signal readout, and secondary confirmation across different functional modules. In addition, related SERS-based biomarker systems further support this design logic. For example, dual-biorecognition strategies combining MIP cavities with antibodies have also been reported for carcinoembryonic antigen detection, where the two recognition elements operate sequentially to provide an additional layer of confirmation. This type of design likewise supports the value of distributing capture/recognition and signal-readout functions across different modules, rather than relying on a single recognition interface alone [102].

### 3.5. Catalytic–Recognition Synergy: Catalytic Gain at the Interface Coupled with Imprint-Based Selectivity

This strategy introduces nanomaterials with electrocatalytic activity, enzyme-mimicking effects, or high conductivity to construct a signal-amplifying interface, which is then coupled with a molecularly imprinted recognition layer. In this way, interfacial charge transfer and the signal-to-noise ratio are improved, while the imprinted cavities provide structurally complementary selectivity. As a result, more stable and highly sensitive electrochemical readout can be achieved without, or with reduced dependence on, biological enzymes and complex labeling systems. This strategy mainly strengthens signal generation and readout reliability by coupling selective recognition with controlled catalytic amplification.

#### 3.5.1. AuNPs/PTh Conductive–Catalytic Network + MIP: Probe-Free Amplification Synergy

Lai et al. [103] constructed an AuNPs/polythiophene (PTh)-modified glassy carbon electrode (GCE) and formed a PDA-based imprinted layer, using AFP as the template, by electropolymerization. Signal readout was performed by differential pulse voltammetry (DPV) in 10 mM [Fe(CN)_6_]^3−^/^4−^ solution. In this system, PTh and AuNPs jointly provided a highly conductive and stable interfacial support and facilitated charge transfer, thereby creating a low-impedance foundation for effective operation of the imprinted layer. The PDA-based imprinted layer then enabled specific recognition of AFP through the cavities formed after template removal. The sensor showed a linear range of 0.001–800 ng/mL, a detection limit of 0.8138 pg/mL, and recoveries of 100.6–111.1% in spiked human serum. This example suggests that when the imprinted layer itself, such as PDA, may introduce additional interfacial impedance or diffusion limitations, it is advantageous to first establish a conductive polymer/gold nanoparticle composite underlayer to reduce charge-transfer resistance before constructing a thin imprinted film. At the same time, classical reversible probes such as [Fe(CN)_6_]^3−^/^4−^ can provide a stable means of converting current or impedance changes induced by binding events into measurable signals, facilitating reuse and comparison across different protein-template systems.

#### 3.5.2. ND/CFNE Electrocatalytic Substrate + Surface-Electropolymerized MIP: In Situ Nanoelectrode Synergy

Zhou et al. [104] introduced nanodiamonds (ND) onto carbon fiber nanoelectrodes (CFNE) and formed an MIP functional layer by electropolymerization for in situ electrochemical detection of DA in living cells. The ND/CFNE platform increased the dopamine (DA) oxidation current by nearly fourfold, greatly improving signal-to-noise ratio and sensitivity. The MIP layer suppressed interference from intracellular structural analogs and enabled specific capture. This nanoelectrode allowed DA measurements at different cytoplasmic locations within single PC12 cells, with a reported cytoplasmic DA concentration of about 0.4 μM. This case suggests that, in nanoelectrode-based or microvolume detection scenarios, the amplification unit should preferably be constructed as a stable and firmly anchored electroactive nanolayer, such as ND, before applying the thinnest possible and well-controlled electropolymerized imprinted layer. Such a design helps balance signal amplification, recognition selectivity, operability in cellular and other complex microenvironments, and repeatable insertion-based measurements.

#### 3.5.3. MXene@AuNPs Catalytic Substrate + Poly(thionine)–MIP: Self-Amplified Detection Synergy

Manikandan et al. [105] constructed an MXene-layered interface with electrodeposited AuNPs on a screen-printed carbon electrode (SPCE), denoted Mx@Au/SPCE. They then carried out in situ electropolymerization using thionine as the functional monomer and creatinine as the template to form the Mx@Au/PTH/MIP structure. The two-dimensional MXene layers provided additional active sites and favorable pathways for electron transfer, while the AuNPs further enhanced the electrochemical response. The overlying poly(thionine)-based imprinted film supplied both selective recognition and an electrochemically readable signal. The system showed a linear range of 0.4–5000 μg/L for creatinine, with a detection limit of 0.03 μg/L (R^2^ = 0.9991), and was applied to rat serum and serum from an adenine-induced renal fibrosis model. This case suggests that, when highly sensitive detection is desired with minimal reliance on external enzymes or complex labels, a high-response substrate can be constructed by combining two-dimensional conductive materials such as MXene with metal nanoparticles. Functional monomers such as thionine, which possess both redox activity and electropolymerization capability, are particularly attractive for building imprinted films because they allow interfacial amplification and recognition-layer readout to be integrated into a single platform. Such a design is readily extendable to other small-molecule metabolites.

#### 3.5.4. MoS_2_/PtNPs Conductive–Catalytic Substrate + Loaded MIP: Synergy of Catalytic Gain and Imprinted Recognition

Mahobiya et al. [106] constructed a MoS_2_/PtNPs composite interface on a screen-printed electrode (SPE), and an MIP for glycated albumin (GA) was prepared by bulk polymerization and then loaded onto the modified surface, yielding MIP/PtNPs/MoS_2_/SPE. The high surface area of two-dimensional MoS_2_ and the catalytic and highly conductive properties of PtNPs jointly enhanced interfacial charge transfer and signal output, while also providing a stable support for the MIP and increasing the density of effective binding sites. Signal readout was carried out by electrochemical impedance spectroscopy (EIS) and square-wave voltammetry (SWV), with a detection range of 0.34 nM–700 μM and a detection limit of 0.34 nM. The sensor showed good selectivity against common serum interferents such as albumin, glycated hemoglobin, and ascorbic acid. After storage at 4 °C under dry conditions for 3 months, about 80% of its activity was retained. This example suggests that, for protein-like biomarkers such as GA in the presence of large molecules and complex backgrounds, improved usability depends not simply on achieving higher current output but on first constructing a stable and high-response substrate through the complementary functions of MoS_2_, which contributes surface area and interfacial transport, and PtNPs, which contribute conductivity and catalysis, and then introducing MIP-based selective recognition. As this study also shows, the transition from measurable performance to practical and reproducible use should be supported by quantitative evidence, including signal loss caused by interferents and long-term stability data.

### 3.6. Interface-Regulation Synergy: Enhancing MIP–Electrode Anchoring and Durability

Conventional MIP films often rely on physical adsorption or weak interfacial interactions for immobilization on electrode surfaces. As a result, partial delamination may occur during template elution, rebinding, or cleaning, which disrupts interfacial charge-transfer pathways and eventually leads to baseline drift and signal attenuation. Interface-regulation synergy addresses this issue by introducing conductive adhesive underlayers, such as PEDOT, bifunctional molecular bridges, such as 4-ATP, or covalent immobilization routes based on surface activation and silanization/crosslinking, such as APTES–glutaraldehyde systems. In this way, interfacial bonding strength can be improved without significantly sacrificing electron transport, leading to more reliable long-term operation and better manufacturability. However, when evaluating the effectiveness of interface-regulation strategies, one should not rely on analytical performance alone but also consider quantifiable indicators such as film integrity after immobilization, baseline stability, reproducibility, and storage durability.

#### 3.6.1. PEDOT/4-ATP Molecular Bridge: Synergy of Interfacial Adhesion Enhancement and Signal Stability

For amyloid β42 (Aβ42) detection, Pereira et al. [107] used a paper-based carbon-ink electrode (CI-HME) as the substrate. PEDOT was first deposited, followed by formation of an interfacial coupling layer using 4-aminothiophenol (4-ATP), and finally an MIP layer was prepared by electropolymerization. PEDOT served as a conductive underlayer that improved the electrochemical features of the paper-based electrode, as evidenced by the appearance of clear redox peaks, very low impedance, and improved surface homogeneity after pretreatment. 4-ATP acted as a molecular bridge: its thiol group was expected to interact with the conductive EDOT/PEDOT layer, while its amino group was proposed to facilitate covalent coupling to the poly(o-phenylenediamine) (PoPD/OPDA) film. As a result, interlayer adhesion was markedly strengthened, and the film stability was improved, as the authors reported that without 4-ATP, the surface became unstable, and the electrode would be unusable. The interfacial relevance of this design was further supported during template-removal analysis: after trypsin/acid treatment, the variation in charge-transfer resistance (Rct) was approximately 30% for the MIP and 13% for the NIP, while CV peak currents recovered after template removal, indicating that the modified interface remained electrochemically responsive during film restructuring and peptide release. Taken together, these observations suggest that the molecular bridge plays a functionally important role in maintaining film integrity, rather than serving as a merely secondary surface additive. The sensor showed a linear range of 0.1 ng/mL to 1 μg/mL. Successful calibration over the same concentration range was also demonstrated in serum, and repeated measurements showed less than 10% variation. This case suggests a transferable interface-regulation principle: for electropolymerized MIP systems prone to delamination, anchoring performance may be more effectively improved by combining a conductive underlayer that enhances electrical continuity and surface uniformity with a bifunctional molecular bridge that reinforces interlayer coupling, rather than relying on the imprinted polymer layer alone to maintain film stability.

#### 3.6.2. MWCNTs/AuNPs + 4-ATP Molecular Bridge + nanoMIPs: Composite Anchoring for Improved Batch-to-Batch Consistency

Akkapinyo et al. [108] constructed a conductive composite interface on an SPCE by combining multi-walled carbon nanotubes (MWCNTs) and AuNPs, followed by 4-ATP-mediated immobilization of nanoMIPs through EDC coupling. SEM and elemental mapping supported stepwise interfacial construction, showing Au deposition and colocalization of Au with sulfur and nitrogen after 4-ATP functionalization, consistent with successful linker immobilization. Electrochemical characterization further showed that Rct decreased from 1258 to 120 Ω after MWCNT modification and to 68 Ω after Au deposition but increased to 118 Ω after 4-ATP functionalization and to 594 Ω after nanoMIP immobilization, reflecting progressive formation of a conductive yet functionalized interface. The resulting sensor exhibited a linear range of 1–100 U/mL and a detection limit of 0.14 U/mL. The nanoMIP system also showed approximately fivefold higher sensitivity than nanoNIP, together with recoveries of 98–102% in human serum and RSD values of 0.52–1.39%. More importantly, several immobilization-related parameters were systematically optimized. Time-dependent results showed that the response associated with 4-ATP coverage and nanoMIP immobilization increased markedly at earlier stages but approached a near-plateau after about 3 h, indicating that further incubation produced only marginal additional changes under these conditions. Therefore, 3 h was considered a practical immobilization time, while EDC concentration was effective up to about 25 mg/mL, beyond which little additional signal change was observed. These results indicate that, in this system, interface chemistry functioned not merely as a passive adhesion step but as an adjustable fabrication variable that influenced nanoMIP loading and electron-transfer behavior. Further, for nanoparticle-immobilized MIP systems, a modular strategy that combines a conductive nanobase, a molecular linker, and controllable covalent coupling can provide a practical route to balancing anchoring stability, interfacial conductivity, and preparation reproducibility.

#### 3.6.3. Nitrogen Plasma Activation + APTES Silanization + Covalent Immobilization of nanoMIPs: Synergy of Long-Term Stability and Anti-Detachment

**Figure 3:**
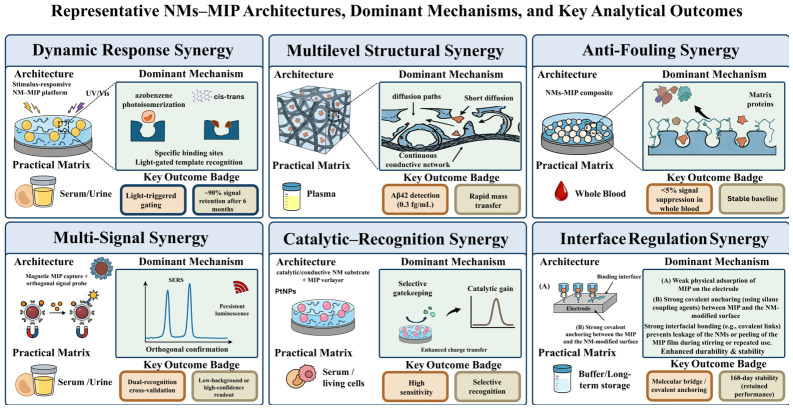
Representative NM–MIP Designs and Key Outcomes.

Cruz et al. [109] prepared electro-responsive nanoMIPs by a solid-phase method for insulin detection and immobilized them onto screen-printed platinum electrodes. The key steps in interfacial modification were nitrogen plasma activation of the electrode surface, followed by silanization with 3-aminopropyltriethoxysilane (APTES) to introduce surface amine groups, and finally covalent fixation of the nanoMIPs via glutaraldehyde crosslinking. In DPV measurements, the sensor showed a linear range of 50–2000 pM, with detection limits of 26 fM in buffer and 81 fM in human plasma, and retained measurable performance after 168 days of storage. Intra-electrode repeatability gave an RSD of 4.2%, while inter-electrode reproducibility gave an RSD of 5.6%. This case shows that surface activation and covalent immobilization can greatly reduce the risks of desorption and signal drift, thereby supporting long-term deployment of nanoMIP-based sensors. For nanoimprinted particle-immobilized systems, a stepwise strategy consisting of plasma activation to increase surface reactivity, APTES silanization to introduce a universal amine-rich primer layer, and glutaraldehyde crosslinking to form a covalent network can substantially improve anti-detachment performance and long-term stability. At the same time, incorporation of electroactive units into nanoMIPs can reduce dependence on external mediators, thereby simplifying the sensing format and making it more suitable for real samples and long-term applications. Representative designs and key outcomes across the six synergistic strategies are summarized in Figure 3.

### 3.7. Integrated Design Principles

Taken together, the six synergistic strategies discussed above suggest that performance enhancement in NM–MIP systems does not arise from isolated, single-point improvement but from modular optimization directed at three key coupled bottlenecks: (i) mass transfer and site accessibility, namely whether recognition sites are sufficiently exposed and diffusion pathways sufficiently short; (ii) signal generation and readout reliability, namely whether charge transfer and amplification mechanisms are controllable and whether the readout provides cross-validation; and (iii) biointerfacial stability and durability, including anti-fouling performance, resistance to delamination, batch-to-batch consistency, and storage stability. From this perspective, the six strategies can be regarded as combinable functional modules. Multilevel structural synergy and dynamic response synergy mainly address mass transfer and kinetic optimization; catalytic–recognition synergy and multi-signal synergy mainly improve signal generation and interpretation reliability; and anti-fouling synergy, together with interface-regulation synergy, forms the practical foundation for operation in complex matrices by governing background noise, signal drift, and long-term usability.

In practical design, integration of these strategies should follow an engineering sequence of foundation first, enhancement later; pathway first, amplification later; and validation first, stacking later. For complex matrices such as serum and plasma, anti-fouling layers and interfacial anchoring layers should be introduced first to reduce the risks of non-specific adsorption and film detachment. When rapid responses to trace biomarkers are required, multilevel structural designs can then be used to shorten diffusion pathways and improve site accessibility. If further improvement in signal-to-noise ratio or reduction in misinterpretation risk is needed, catalytic amplification modules and dual-/multi-mode cross-validation can be incorporated to strengthen result reliability. For continuous monitoring or wearable applications, dynamic response units should be prioritized to improve reversible binding and resistance to environmental disturbances. It is important to emphasize that synergy is not equivalent to simply stacking more layers: excessive layering or increased film thickness may introduce mass-transfer limitations, increase interfacial resistance, and reduce compatibility, thereby offsetting gains in sensitivity or response speed.

**Figure 4:**
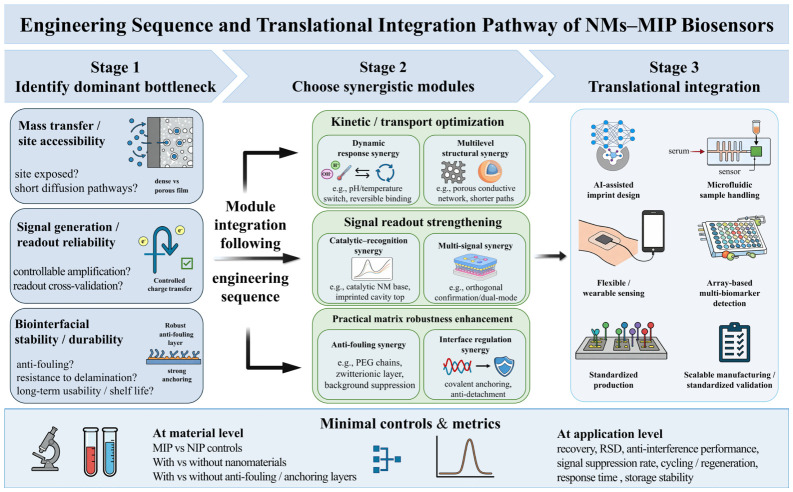
Design Roadmap for NM–MIP Biosensors.

To ensure that conclusions regarding synergy are both reproducible and transferable, attribution analysis should be performed using a minimal set of controls and a clear metric system. At the material level, controls should include MIP versus NIP, with versus without nanomaterials, and with versus without anti-fouling or anchoring layers. At the application level, key parameters such as recovery, relative standard deviation (RSD), anti-interference performance, cycling/regeneration stability, storage stability, and, where appropriate, signal-suppression rate and response time should be reported. Performance gains should then be mapped explicitly to the dominant bottleneck addressed, such as mass transfer, electron transfer, background suppression, or interfacial durability. Accordingly, rather than stacking functional materials, effective NM–MIP biosensor design should follow a mechanism-oriented integration logic, where each functional module is deliberately introduced to address a specific bottleneck. The design logic for strategy integration is summarized in Figure 4, and broader strategy–performance comparisons are compiled in Table 1.

## 4. Challenges and Future Outlook

Although NM–MIP synergistically modified electrochemical sensors have achieved significant progress in enhancing sensitivity and selectivity, an important challenge for further development lies not only in achieving better analytical performance but also in whether the reported synergistic designs can provide sufficiently reliable and practically transferable solutions for biomarker analysis. This is particularly true in addressing clinical demands such as reliable interpretation of detection results in complex biological matrices and long-term stable operation [117,118]. As discussed in the preceding sections, several recurring issues remain insufficiently resolved, particularly the incomplete separation of different performance contributions, limited robustness evaluation in complex samples, fabrication reproducibility, and the difficulty of integrating high analytical performance with simplified and application-compatible formats.

In many reported systems, performance gains are still inferred mainly from endpoint analytical results, whereas the respective contributions of site accessibility, interfacial charge transfer, anti-fouling behavior, and anchoring stability are not always clearly distinguished. Meanwhile, matrix tolerance and workflow simplification remain major bottlenecks for practical use. Non-specific adsorption (biofouling) induced by complex biological matrices readily causes baseline drift, signal attenuation, and reduced reproducibility, thereby increasing the risk of misdiagnosis [119]. To address this challenge, strategies include constructing hydrophilic/amphoteric anti-contamination interface layers, employing surface imprinting or film formation to enhance site exposure, and integrating conductive/porous nanoscale scaffolds to mitigate contamination’s adverse effects on recognition and charge transfer [120]. At the same time, these effects should ideally be evaluated through matched comparisons between buffer and real samples, rather than being inferred only from successful operation in complex matrices.

Against this background, future technical development is more usefully considered in terms of how it may address these shared bottlenecks, rather than as a separate list of emerging concepts. Traditional MIP synthesis still relies heavily on trial-and-error optimization of functional monomers, crosslinkers, and polymerization conditions. To improve design efficiency and reduce empirical screening, artificial intelligence (AI), computational simulation, and machine-learning-assisted strategies are increasingly being introduced into MIP research. Existing studies have shown that machine learning can be used to predict imprinting quality and assist in monomer selection or the optimization of key synthesis parameters [121,122,123]. Building on these advances, AI-assisted design may be further extended to the nanomaterial–MIP integration level. In such systems, variables such as nanomaterial type and loading, linker chemistry, film thickness, and interfacial assembly sequence are often interdependent and may therefore be better optimized in a coordinated rather than isolated manner. In this context, a possible framework would combine molecular-level screening, interfacial parameter selection, and iterative experimental feedback to support more rational design of integrated nanomaterial–MIP architectures [124,125]. Furthermore, integrating NM–MIP with microfluidics, particularly paper-based microfluidics, enables automated sample introduction, separation, and washing. This approach can help reduce non-specific adsorption at the device-design level and is therefore more compatible with point-of-care testing (POCT) requirements. Moreover, for dynamic physiological monitoring, stable MIP recognition layers must be integrated with flexible substrates, miniaturized electrochemical readout modules, and wireless data transmission systems [126,127,128,129]. Recent wearable prototypes have begun to demonstrate the feasibility of continuous or in situ analysis of glucose and biomarkers in sweat [130,131,132].

**Figure 5:**
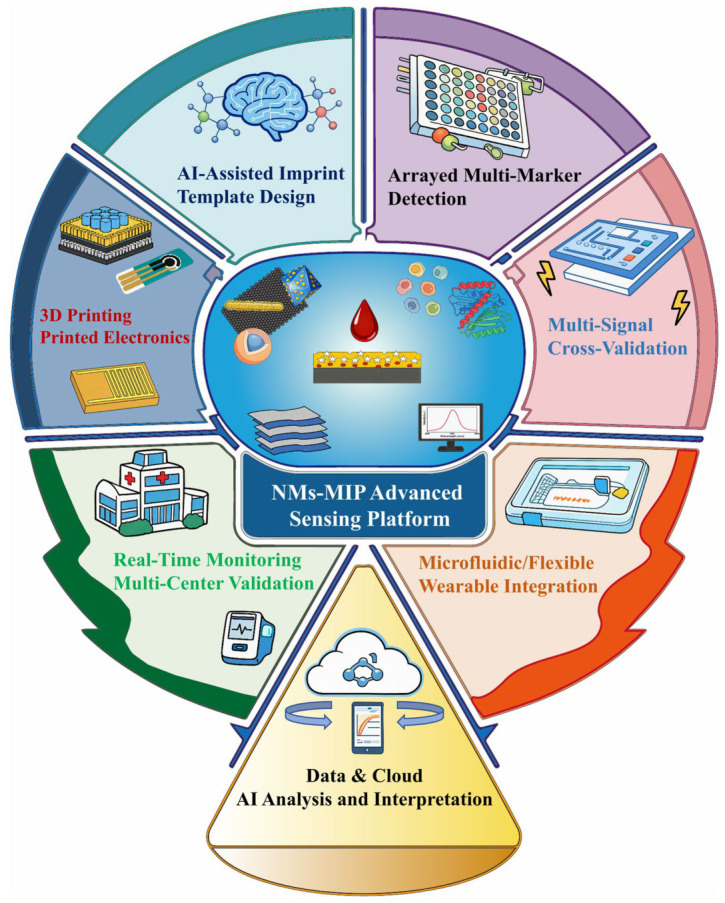
Integration of NM–MIP Sensors with Emerging Technologies.

Overall, NM–MIP sensors still face significant challenges in clinical translation and commercialization [133], including consistent large-scale fabrication [134], high-specificity detection of low-abundance biomarkers in complex samples, insufficient validation of biocompatibility and stability for long-term in vivo/ex vivo applications, lack of large clinical datasets, and absence of standardized processes meeting regulatory requirements. Accordingly, future progress will likely depend on coupling synergistic material design with technologies that better support scalable fabrication, integrated sample handling, and portable operation. In this regard, AI and computational simulation [135,136,137], microfluidics, and flexible wearable technologies [138,139] are particularly relevant because they may improve design efficiency, workflow integration, and compatibility with practical sensing formats. Furthermore, emerging technologies such as array-based multi-marker detection, 3D printing, printed electronics and micro/nanofabrication, and multimodal cross-validation, though not yet systematically validated in NM–MIP electrochemical sensing systems, warrant exploration for their potential synergistic roles across functional modules. Relevant technical concepts are illustrated in Figure 5. Taken together, these six synergistic strategies can be viewed as a transferable, mechanism-oriented reference framework for selecting and integrating functional modules in NM–MIP biosensors according to different sensing bottlenecks and application needs.

In summary, future research should focus on advancing functional nanomaterial innovation, intelligent design and miniaturized system integration, automated and scalable manufacturing and expanding applications across diverse scenarios [140,141]. Through multidisciplinary integration and technological convergence, NM–MIP electrochemical sensors hold promise to further enhance their translational potential in early disease diagnosis and precision medicine, providing more accurate, rapid, and convenient analytical tools for clinical testing.

## Figures and Tables

**Table 1:** Representative NM–MIP biosensors for biomarker detection: compositions, synergistic strategies, readout modes, and key analytical performance.

Author (Year)	Target Biomarker	Practical Application Samples	NM–MIP Sensor Composition	Core Synergistic Strategy	Detection Method	Detection Limit	Linear Range
Wang et al. (2021) [89]	EV71	Human serum	Photo-magnetic dual-response MIP (Fe_3_O_4_@SiO_2_@MOF)	Dynamic Response Synergy	RLS	9.5 × 10^−3^ U/mL	0.05–5.0 U/mL
Luo et al. (2020) [90]	HAV	Human serum	MIL-101-supported pH-responsive HM@MIPs	Dynamic Response Synergy	RLS	0.1 pmol/L	0.02–2.0 nmol/L
Zhao et al. (2020) [91]	BHb	urine	CD@SiO_2_@MIP with NIPAAm/PNIPAAm thermoresponsive fluorescent platform	Dynamic Response Synergy	Fluorescence	0.155 μM	0.31–15.5 μM
Taheri et al. (2022) [92]	CEA; AFP	Human serum	Rectangular nanotubular double-template PPy–MIP/FTO electrode	Multilevel Structural Synergy	EIS	AFP: 3.3 pg/mL; CEA: 1.6 pg/mL	AFP: 10–10^4^ pg/mL; CEA: 5–10^4^ pg/mL
Pakapongpan et al. (2024) [93]	Aβ42	Buffer/Simulated Sample	Nitrogen-doped carbon dots–graphene/PPy–MIP screen-printed electrode	Multilevel Structural Synergy	DPV	1 pg/mL	5–70 pg/mL
Saddique et al. (2023) [94]	Creatinine	Saliva	Schlegelite (MoO_3_/V_2_O_5_) nanocrystals/MIP electrochemical electrode	Multilevel Structural Synergy	DPV	90 nM	5–30 μM
Ait Lahcen et al. (2021) [95]	HER–2	Human serum	Laser-etched graphene/nanogold/PEDOT–MIP	Anti-fouling Synergy	DPV	0.43 ng/mL	1–200 ng/mL
Li et al. (2024) [96]	Creatinine; Urea; HSA	Human serum; urine	rGO/PDA surface-imprinted multi-marker MIP platform	Anti-fouling Synergy	DPV	Creatinine, 0.27 ± 0.01 fg/mL; Urea, 3.87 ± 0.13 fg/mL; HSA, 0.52 ± 0.02 fg/mL	Creatinine, 10^2^–10^12^ fg/mL; Urea, 10^2^–10^12^ fg/mL; HSA, 10^0^–10^8^ fg/mL
Yang et al. (2024) [97]	SARS-CoV-2 spike protein	Human serum	PPy-based surface-imprinted ratiometric biosensor with SA on boronate-affinity AuNPs/SPCE	Anti-fouling Synergy	DPV	30.1 fg mL^−1^	100 fg mL^−1^–100 ng mL^−1^
Zhu et al. (2025) [98]	Glucose	Whole blood	Ni_3_(HITP)_2_-MOF/MIP/PTB anti-fouling barrier electrode	Anti-fouling Synergy	DPV	0.31 μM	1 μM–100 mM
Zhang et al. (2024) [100]	DA	Serum; sweat; urine	Fe_3_O_4_-based m-MIPs + ZGGC-Apt persistent-luminescence probe	Multi-signal Synergy	Persistent luminescence/fluorescence	0.0065 μM	0.01–100 μM
Turan et al. (2022) [101]	PSA	Human serum	Magnetic MIP capture probe + Au–SERS label	Multi-signal Synergy	SERS	0.9 pg/mL	0.5 pg/mL–1.0 μg/mL
Lai et al. (2019) [103]	AFP	Human serum	AuNPs/PTh-modified GCE + PDA-MIP	Catalytic-Recognition Integrated Synergy	DPV	0.8138 pg/mL	0.001–800 ng/mL
Zhou et al. (2024) [104]	DA	Living cells (single PC12 cells)	ND/CFNE with surface-electropolymerized MIP layer	Catalytic–Recognition Integrated Synergy	DPV	5 nM	0.1–5.0 μM
Manikandan et al. (2025) [105]	Creatinine	Rat serum	SPCE/MXene@AuNPs/PTH-MIP	Catalytic-Recognition Integrated Synergy	DPV	0.03 μg·L^−1^	0.4–5000 μg·L^−1^
Mahobiya et al. (2022) [106]	GA	Human serum	MoS_2_/PtNPs Conductive–Catalytic Substrate + MIP Powder Loading	Catalytic-Recognition Integrated Synergy	EIS/SWV	0.34 nM	0.34 nM–35 μM; 200–700 μM
Pereira et al. (2020) [107]	Aβ42	Human serum	CI-HME/PEDOT/4-ATP/PoPD-MIP	Interface-Regulation Synergy	SWV	0.067 ng/mL	0.1 ng/mL–1 μg/mL
Akkapinyo et al. (2025) [108]	CA15-3	Human serum	nanoMIPs/4-ATP/Au/MWCNTs/SPCE	Interface-Regulation Synergy	SWV	0.14 U/mL	1–100 U/mL
Cruz et al. (2020) [109]	Insulin	Human plasma	Electro-responsive nanoMIPs immobilized on screen-printed platinum electrode via nitrogen plasma activation/APTES/glutaraldehyde	Interface-Regulation Synergy	DPV	81 fM	50–2000 pM
Pirzada et al. (2020) [110]	NSE	Human serum	AuNP-decorated dual-epitope-mediated hybrid MIP electrochemical sensor	Catalytic–Recognition Integrated Synergy	SWV	25 pg/mL	25–500 pg/mL
Oliveira et al. (2023) [111]	CA15–3	Human serum	AuNPs/PoPD–MIP-modified disposable paper-based electrode	Anti-fouling Synergy	DPV	1.16 U/mL	5–35 U/mL
Özcan et al. (2020) [112]	Aβ42	Human plasma	MXene/MWCNTs/PPy–MIP-modified glassy carbon electrode	Multilevel Structural Synergy	DPV	0.3 fg/mL	1.0–100.0 fg/mL
Almehizia et al. (2023) [113]	Myoglobin	Artificial serum	MIP/MWCNT composite membrane-modified paper-based potentiometric sensor	Multilevel Structural Synergy	Potentiometric	28 nM	5.0 × 10^−8^–1.0 × 10^−4^ M
Antipchik et al. (2022) [114]	HCV E2	Human plasma	Cuttable interlayer/PmPD–MIP-modified screen-printed electrode	Interface-Regulation Synergy	DPV	4.6 × 10^−4^ ng/mL	0.01–50 ng/mL
Zhao et al. (2020) [115]	BHb	Urine	CD@SiO_2_–PNIPAAm	Dynamic Response Synergy	Fluorescence	0.155 μM	0.31–15.5 μM
Bi et al. (2020) [116]	KAN	Milk, water samples	Au@Fe_3_O_4_ tracking label + aptamer/MIP dual-recognition electrode	Multi-signal Synergy	DPV	1.87 nM	10–500 nM

## Data Availability

No new data were created or analyzed in this study.

## Abbreviations

The following abbreviations are used in this manuscript:

Aβ42	amyloid beta 42
AI	artificial intelligence
AFP	alpha-fetoprotein
Apt	aptamer
ATRP	atom transfer radical polymerization
CLIA	chemiluminescent immunoassay
CV	cyclic voltammetry
DA	dopamine
DPV	differential pulse voltammetry
EIS	electrochemical impedance spectroscopy
ELISA	enzyme-linked immunosorbent assay
EV71	enterovirus 71
GA	glycated albumin
GCE	glassy carbon electrode
HAV	hepatitis A virus
HCV	hepatitis C virus
KAN	kanamycin
LC–MS/MS	liquid chromatography–tandem mass spectrometry
LOD	limit of detection
MIP	molecularly imprinted polymer
MIT	molecular imprinting technology
MOF	metal–organic framework
MXene	2D transition metal carbide/nitride
MWCNTs	multi-walled carbon nanotubes
NIP	non-imprinted polymer
NM	nanomaterial
NM–MIP	nanomaterial–molecularly imprinted polymer
o-PD	o-phenylenediamine
PCR	polymerase chain reaction
PEDOT	poly(3,4-ethylenedioxythiophene)
PEI	polyethyleneimine
POCT	point-of-care testing
PPy	polypyrrole
Py	pyrrole
PTB	phase-transformed bovine serum albumin
RLS	resonant light scattering
Rct	charge-transfer resistance
SERS	surface-enhanced Raman scattering
SPCE	screen-printed carbon electrode
SWV	square wave voltammetry
